# Quantifying Climatological Ranges and Anomalies for Pacific Coral Reef Ecosystems

**DOI:** 10.1371/journal.pone.0061974

**Published:** 2013-04-18

**Authors:** Jamison M. Gove, Gareth J. Williams, Margaret A. McManus, Scott F. Heron, Stuart A. Sandin, Oliver J. Vetter, David G. Foley

**Affiliations:** 1 Joint Institute for Marine and Atmospheric Research, University of Hawaìi at Mānoa, Honolulu, Hawaíi, United States of America; 2 Coral Reef Ecosystem Division, NOAA Pacific Islands Fisheries Science Center, Honolulu, Hawaíi, United States of America; 3 Scripps Institution of Oceanography, University of California San Diego, San Diego, California, United States of America; 4 School of Ocean and Earth Science and Technology, University of Hawaìi at Mānoa, Honolulu, Hawaíi, United States of America; 5 Coral Reef Watch, NOAA National Environmental and Satellite, Data, and Information Service, Silver Spring, Maryland, United States of America; 6 Marine Geophysical Laboratory, Physics Department, School of Engineering and Physical Sciences, James Cook University, Townsville, Queensland, Australia; 7 Environmental Research Division, NOAA Southwest Fisheries Science Center, Pacific Grove, California, United States of America; The Australian National University, Australia

## Abstract

Coral reef ecosystems are exposed to a range of environmental forcings that vary on daily to decadal time scales and across spatial scales spanning from reefs to archipelagos. Environmental variability is a major determinant of reef ecosystem structure and function, including coral reef extent and growth rates, and the abundance, diversity, and morphology of reef organisms. Proper characterization of environmental forcings on coral reef ecosystems is critical if we are to understand the dynamics and implications of abiotic–biotic interactions on reef ecosystems. This study combines high-resolution bathymetric information with remotely sensed sea surface temperature, chlorophyll-*a* and irradiance data, and modeled wave data to quantify environmental forcings on coral reefs. We present a methodological approach to develop spatially constrained, island- and atoll-scale metrics that quantify climatological range limits and anomalous environmental forcings across U.S. Pacific coral reef ecosystems. Our results indicate considerable spatial heterogeneity in climatological ranges and anomalies across 41 islands and atolls, with emergent spatial patterns specific to each environmental forcing. For example, wave energy was greatest at northern latitudes and generally decreased with latitude. In contrast, chlorophyll-*a* was greatest at reef ecosystems proximate to the equator and northern-most locations, showing little synchrony with latitude. In addition, we find that the reef ecosystems with the highest chlorophyll-*a* concentrations; Jarvis, Howland, Baker, Palmyra and Kingman are each uninhabited and are characterized by high hard coral cover and large numbers of predatory fishes. Finally, we find that scaling environmental data to the spatial footprint of individual islands and atolls is more likely to capture local environmental forcings, as chlorophyll-*a* concentrations decreased at relatively short distances (>7 km) from 85% of our study locations. These metrics will help identify reef ecosystems most exposed to environmental stress as well as systems that may be more resistant or resilient to future climate change.

## Introduction

Coral reef ecosystems are exposed to a suite of physical, chemical and biological environmental forcings that are highly variable across time and space [1], [2]. Environmental forcings influence coral reef ecosystem process and function, including coral reef extent and growth rates and the abundance, diversity, and morphology of reef organisms [1]. Over time, coral reefs have adapted to exist within a particular climatological range; an envelope of environmental forcings that is region-specific and governed by a reef's geographic location [2], [3]. Regional variation (hundreds to thousands of kilometers) in the climatological range is a major determinant of spatial differences in coral reef communities [1] and how they respond to environmental forcings [4]. Anomalous environmental forcings exceed the climatological range limits and are considered beyond a reef ecosystem's ‘normal’ or adapted range of environmental conditions [3]. Anomalous environmental forcings have caused mass coral mortality and shifts in reef community structure [5]–[7], even in the most remote parts of the world [8]–[10].

Previous research has focused on the characterization of environmental forcings that influence coral reef ecosystems across broad geographic areas [3], [11]–[14]. Such studies have been important for establishing environmental limits to coral reef development [3], identifying broad geographic patterns in environmental habitats in which coral reefs reside [13], [15], and assessing the susceptibility of coral reefs to anomalies in environmental forcings on a global scale [11], [12], [16]. In many previous studies, [3], [13], [14] environmental data were synthesized at 1 × 1° (12,100 km^2^); a coarse resolution when compared to the size of many of the islands and atolls in the Pacific. Research has shown environmental conditions such as productivity [17], [18] and temperature [19], [20] proximate to islands can be distinct from regional conditions. Hence, downscaling is needed to better assess environmental forcings at the scale of individual island- and atoll-reef ecosystems.

Work to date that focused on the characterization of environmental conditions in which coral reefs reside has included a broad suite of environmental forcings (i.e. temperature, irradiance, chlorophyll-*a*, nutrients, aragonite saturation state, wind, currents, and sedimentation). However, wave energy, a major environmental forcing determining coral reef community patterns [21]–[25], has been conspicuously absent. Gradients in wave energy and associated flow produce different levels of disturbance which, in turn, lead to changes in benthic community composition and coral morphology [1], [22], [23], [26], [27]. Wave energy can also mix the upper water column, reducing temperatures during warming events [28] and potentially enhancing surface nutrient availability [29]. Although larger, highly episodic wave events (i.e., generated by tropical cyclones) are also of great ecological relevance [7], [30], including the prevailing wave climate would more thoroughly characterize the environmental conditions of coral reef ecosystems [31].

In recent decades it has become clear that coral reef communities are not only structured by natural environmental forcings, but also by human activities [32], [33]. Global-scale forcing associated with human activity is driving ocean warming [34], ocean acidification [35], sea-level rise [36], and increased intensity of tropical cyclones [37], each of which have profound implications for the future of coral reef ecosystems [2], [32], [38]–[42]. The resiliency of corals and their ability to adapt to future environmental conditions may be, in part, linked to their historical environmental climate [9], [16], [43]–[45]. For example, Castillo et al., 2012 [45] showed differences in coral growth based on oceanographic habitat, concluding that corals in more thermally variable environments may be better acclimatized and/or adapted to thermal stress than corals inhabiting more thermally stable environments. Such inductive reasoning may not apply to all environmental forcings. However, assessing a reef ecosystem's environmental setting as well as shorter-term environmental variability will provide insight into how these ecosystems may respond to future climate scenarios.

In this research, we build upon previous studies and present a unique methodological approach to characterize environmental forcings at the scale of individual islands and atolls. We incorporate the following remotely sensed and modeled parameters; sea surface temperature (SST), wave energy, chlorophyll-*a* (proxy for phytoplankton biomass) and irradiance. Previous research has shown these parameters to be among the primary environmental drivers for spatiotemporal differences in coral reef communities [1]–[3], [7], [13], [22], [46], [47]. There are inherent limits to satellite-derived and modeled information [48], [49]; therefore, additional environmental forcings pertinent to coral reef communities (e.g. nutrients, aragonite saturation state, salinity, etc.) are presently unavailable at the resolution (spatial and/or temporal) required to calculate a robust island- and atoll-scale environmental setting for these properties.

Here, we target the U.S.-owned and affiliated reef ecosystems in the Pacific that are the focus of a long-term coral reef ecosystem monitoring program, NOAA's Pacific Reef Assessment and Monitoring Program (Pacific RAMP). Pacific RAMP surveys coral reefs that reside in disparate oceanographic regimes and that are exposed to varying levels of potential human impact. Locations include the heavily populated and urbanized islands of Oahu, Guam, and Saipan, as well as some of the most isolated and pristine reef ecosystems in the Pacific, such as Kingman, Palmyra, Howland, Baker, and Jarvis.

**Figure 1:**
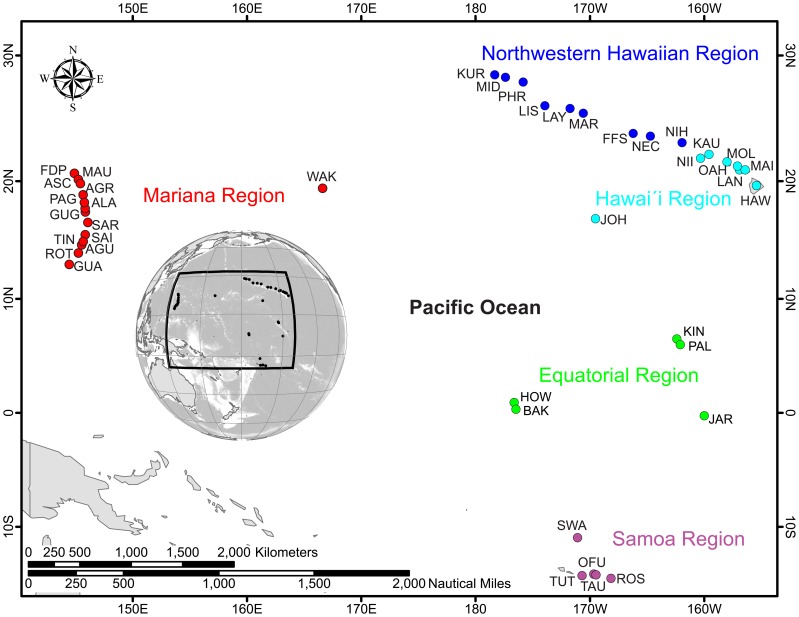
Map highlighting the 41 islands and atolls that comprise the coral reef ecosystems of the U.S. Pacific. Individual locations are color-coded by region. Regions include Northwestern Hawaiian, Hawaíi, Mariana, Equatorial and Samoa. Table 1 provides additional information pertaining to each location, including the location name for each of the location codes.

**Figure 2:**
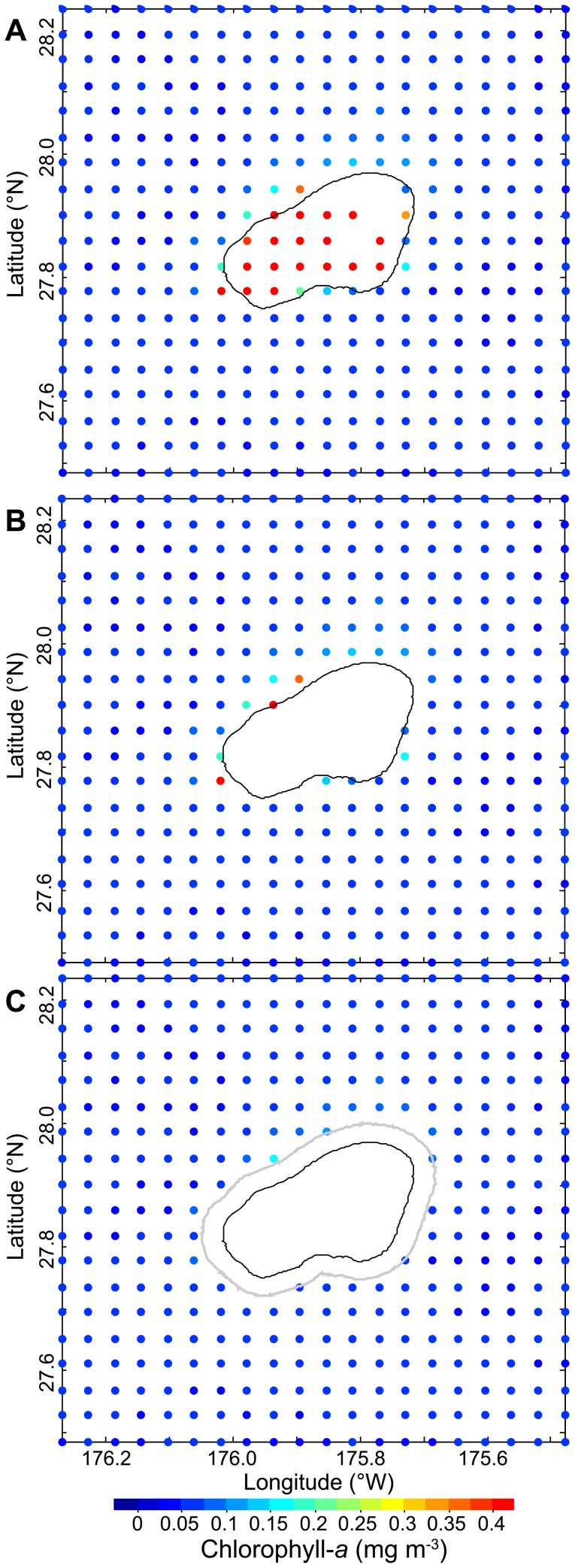
Monthly composite of chlorophyll-*a* concentrations at Pearl and Hermes Reef in the Northwestern Hawaiian Region for September 2003. A) Unfiltered data with contaminated information associated with shallow-water bottom reflectance; B) data filtered using the 30-m bathymetric contour (black line), although contaminated information still remains as a result of bottom reflectance; C) fully cleaned data set using an additional data removal filter (gray line) that is everywhere perpendicular to the 30-m contour, removing all contaminated data associated with bottom reflectance.

**Figure 3:**
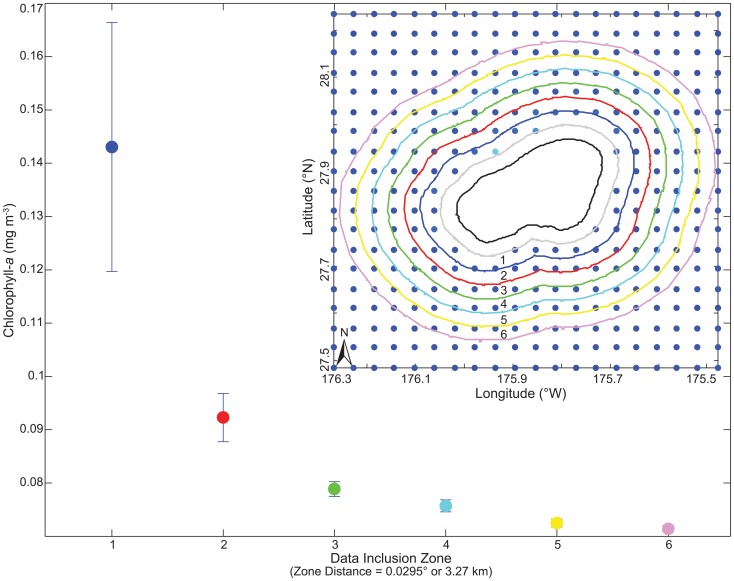
Chlorophyll-a concentrations (mg m^−3^±standard error) by data inclusion zone from the error-free data set (Fig. 2) at Pearl and Hermes Reef. Values for each data inclusion zone were calculated by taking the long-term mean of each pixel from July 2002 to May 2011, and then averaging over all pixels within each zone. The numbers on the *x*-axis are associated with sequentially expanding data inclusion zone, separated by 0.0295° (∼3.27 km). The data inclusion zones are exclusive and nonoverlapping, and color-coded and numbered based on the inset of Pearl and Hermes Reef. The black line represents the 30-m isobath and the gray line represents the additional data removal filter.

**Figure 4:**
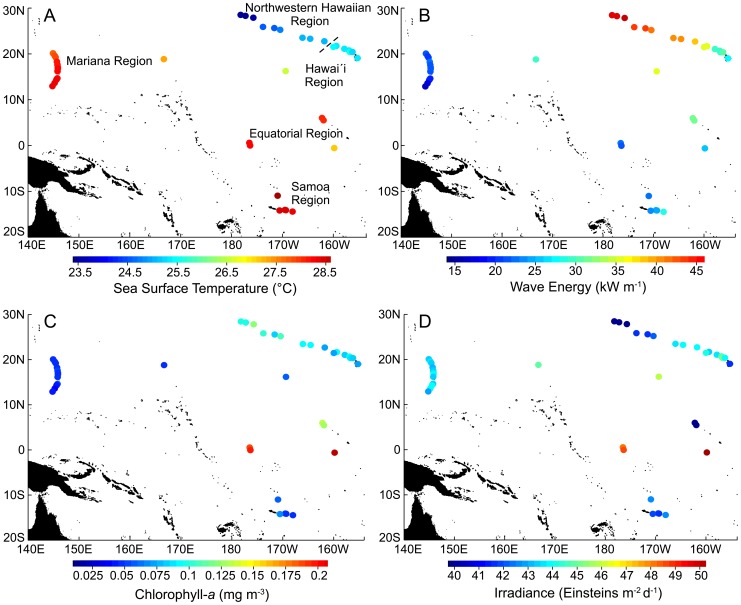
Map of long-term means in A) SST, B) wave energy, C) chlorophyll-*a* and D) irradiance across each of the regions that comprise the coral reef ecosystems of the U.S. Pacific. Regions indicated in panel A are the same for panels B –D. Please see Fig. 1 as a reference for individual island and atoll locations.

**Figure 5:**
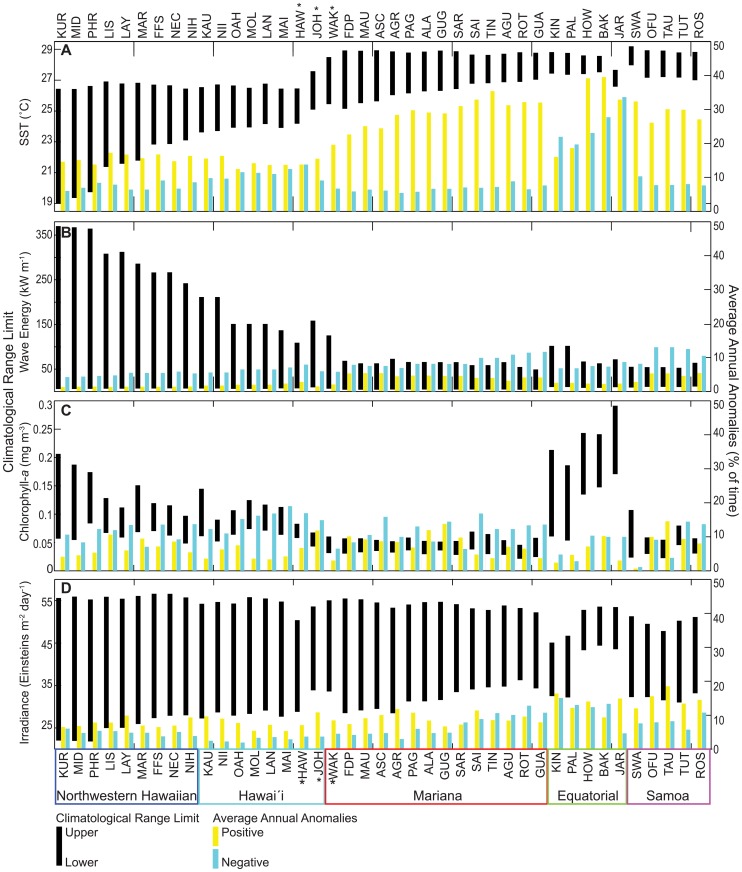
Climatological ranges and average annual anomalies for U.S. Pacific coral reef ecosystems. Island- and atoll-scale metrics for A) SST, B) wave energy, C) chlorophyll-*a* and D) irradiance. In each of the panels (A – D), the black bar represents the climatological range, with the top and bottom of the bar representing the upper and lower climatological range limits, respectively. The yellow and blue bars signify average annual positive and negative anomalies, respectively, represented here as the average annual percentage of time above (positive) the upper climatological range limit and below (negative) the lower climatological range limit. Islands and atoll names are presented as a three-letter code (see Table 1 for full location names), grouped and color-coded by region (see Fig. 1 for map of locations), and oriented by decreasing latitude from left to right (see Table 1 for specific positions). The asterisks represent the islands that are oriented based on geographic proximity to other islands and atolls, as opposed to strict latitudinal orientation. See Tables S1, S2, S3, S4, S5, S6 for climatology and anomaly values presented in this figure.

**Figure 6:**
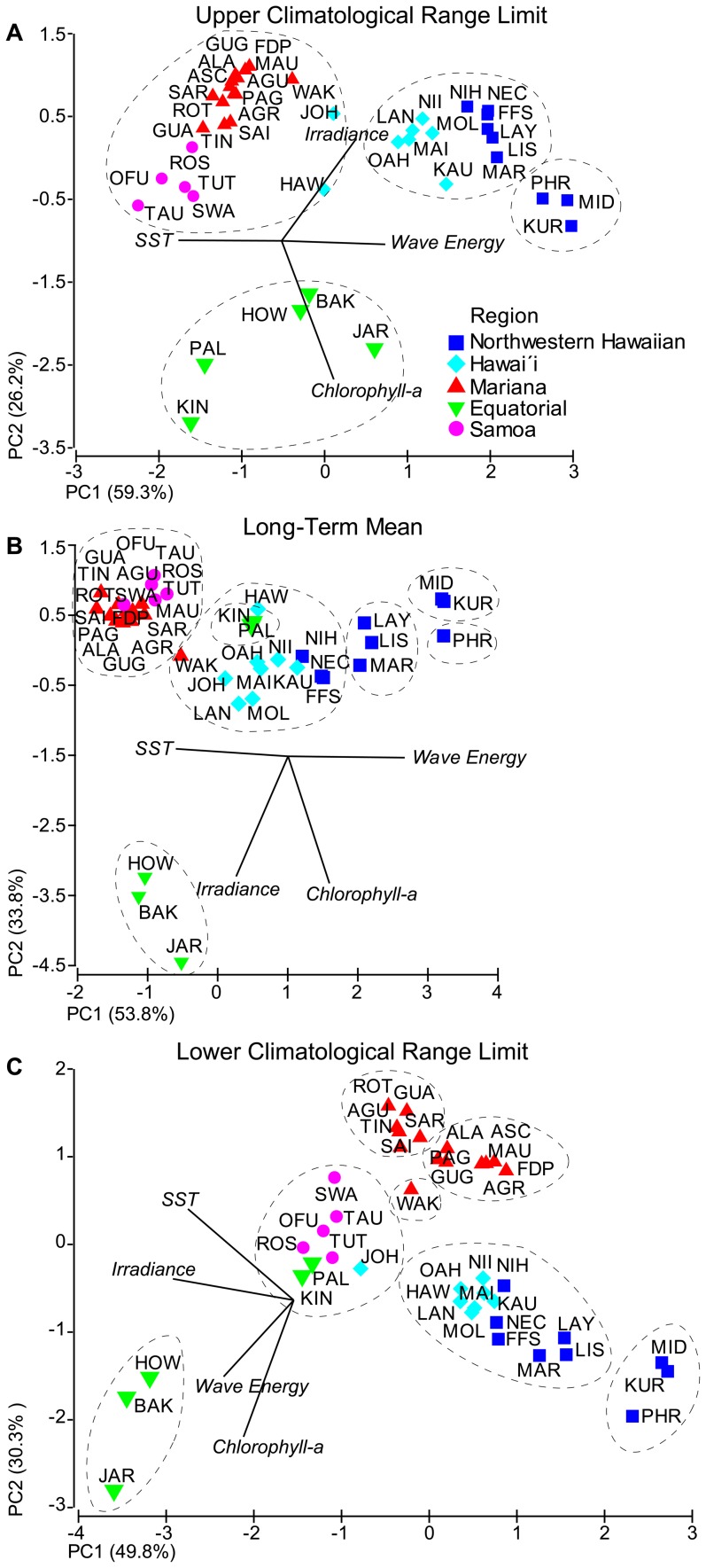
Principle component analysis (PCA) of the A) upper climatological range limit, B) long-term mean and C) lower climatological range limit for all environmental forcings: SST, wave energy, chlorophyll-*a*, irradiance. Island and atoll names are presented as a three-letter code (see Table 1 for full location names) and grouped and color-coded by region (see Fig. 1 for map of locations). The direction of loading for each of the parameters is indicated by the black line, with the direction of the line pointed towards increasing values. Similarity Profile (SIMPROF) results are represented by dashed lines and indicate islands with similar environmental forcings (*p*<0.0001) with respect to each of the metrics. See Table S1 for climatological range limit and long-term mean values presented in this figure.

Using high-resolution bathymetric data, we spatially constrain and quality control environmental data across 41 islands and atolls that comprise the coral reef ecosystems of the U.S. Pacific (Figs. 1, 2, 3; Table 1). We then derive island- and atoll-scale metrics that quantify climatological range limits and the occurrence and magnitude of anomalous events (events that fall outside of the climatologic range) for each environmental forcing at each study location (Tables S1, S2, S3, S4, S5, S6). We then present these metrics from a univariate perspective, to compare the latitudinal differences in climatological ranges and anomalies at all locations (Figs. 4 – 5, S1, S2, S3, S4), and from a multivariate perspective, to more comprehensively assess environmental conditions of reef ecosystems relative to each other (Fig. 6).

**Table 1:** Table of information for each of the 41 islands and atolls that comprise the coral reef ecosystems of the U.S. Pacific.

Island Name	Island Code	Island Type	Latitude	Longitude	Land Area	Reef Area
**Northwestern Hawaiian Region**						
Kure	KUR	Closed atoll	28.42	−178.33	0.92	83.15
Midway	MID	Closed atoll	28.23	−177.38	5.98	101.52
Pearl & Hermes Reef	PHR	Closed atoll	27.86	−175.85	0.50	467.27
Lisianski	LIS	Open atoll	26.01	−173.95	1.50	1004.27
Laysan	LAY	Carbonate island	25.78	−171.73	3.53	488.13
Maro Reef	MAR	Open atoll	25.41	−170.58	0.00	1075.44
French Frigate Shoals	FFS	Open atoll	23.79	−166.21	0.20	677.96
Necker	NEC	Basalt island	23.58	−164.70	0.12	1028.32
Nihoa	NIH	Basalt island	23.06	−161.93	0.72	0.74
**Hawaíi Region**						
Kauai	KAU	Basalt/Carbonate island	22.09	−159.57	1436.70	241.70
Niihau	NII	Basalt/Carbonate island	21.90	−160.15	186.82	108.06
Oahu	OAH	Basalt/Carbonate island	21.49	−158.00	1548.99	422.72
Molokai	MOL	Basalt/Carbonate island	21.14	−157.09	670.22	198.51
Lanai	LAN	Basalt island	20.82	−156.92	365.37	55.49
Maui	MAI	Basalt island	20.82	−156.40	1886.32	196.84
Hawaii	HAW	Basalt island	19.53	−155.42	10441.51	201.67
Johnston	JOH	Open atoll	16.74	−169.52	2.63	194.01
**Mariana Region**						
Wake	WAK	Closed atoll	19.30	166.62	6.97	19.18
Farallon de Pajaros	FDP	Basalt island	20.55	144.89	2.25	1.38
Maug	MAU	Basalt island	20.02	145.22	2.14	3.17
Asuncion	ASC	Basalt island	19.69	145.40	7.86	2.54
Agrihan	AGR	Basalt island	18.76	145.66	44.05	9.50
Pagan	PAG	Basalt island	18.11	145.76	47.75	16.29
Alamagan	ALA	Basalt island	17.60	145.83	12.96	4.28
Guguan	GUG	Basalt island	17.31	145.84	4.24	2.00
Sarigan	SAR	Basalt island	16.71	145.78	4.47	2.00
Saipan	SAI	Basalt/Carbonate island	15.19	145.75	118.98	73.04
Tinian	TIN	Basalt/Carbonate island	14.99	145.63	101.21	16.20
Aguijan	AGU	Basalt/Carbonate island	14.85	145.55	7.01	5.91
Rota	ROT	Basalt/Carbonate island	14.16	145.21	85.13	16.03
Guam	GUA	Basalt/Carbonate island	13.46	144.79	544.34	94.85
**Equatorial Region**						
Kingman	KIN	Open atoll	6.40	−162.38	0.76	47.63
Palmyra	PAL	Closed atoll	5.88	−162.09	2.23	52.50
Howland	HOW	Carbonate island	0.80	−176.62	1.80	2.57
Baker	BAK	Carbonate island	0.20	−176.48	1.60	4.43
Jarvis	JAR	Carbonate island	−0.37	−160.00	4.43	4.32
**Samoa Region**						
Swains	SWA	Carbonate island	−11.06	−171.08	2.38	2.82
Ofu & Olosega	OFU	Basalt island	−14.17	−169.65	12.61	12.03
Tau	TAU	Basalt island	−14.24	−169.47	45.09	10.38
Tutuila	TUT	Basalt island	−14.30	−170.70	137.45	50.89
Rose	ROS	Closed atoll	−14.55	−168.16	0.09	7.80

All locations are grouped by regions, indicated in bold. *Island Name* is the name of the island or atoll. *Island* is the three-letter code used in Figures 1, 5 and 6. *Island Type* is based on primary geological make-up. Closed atoll designation is where a majority of the atoll is enclosed by emergent or semi-emergent reef. *Latitude* and *Longitude* are in degrees north and east, respectively, based on the center point of each island and atoll. *Land Area* and *Reef Area* are shown in square kilometers. *Reef Area* is calculated from the shoreline to the 30-m isobath.

## Materials and Methods

Satellite-derived observations and model output of SST, wave height and period, chlorophyll-*a* and irradiance were used to develop time series data sets and quantify long-term means (Figs. 4, 6B; Table S1) climatological range limits (Figs. 5, 6A, 6C, S1 – S2; Tables S1, S3, S4, S5, S6) and the magnitude and occurrence of anomalous events (Figs. 5, S3 – S4; Tables S2, S3, S4, S5, S6). The following describes the methodological approach to spatially constrain and quality control these data to characterize island and atoll (henceforth referred to as island for both) specific environmental forcing. Additional information pertaining to the quality control of each environmental data set is found in Appendix S1.

*SST*: Derived from the Pathfinder v5.0 dataset (http://pathfinder.nodc.noaa.gov, [50]), we quantified SST using a 0.0439° (hereafter 4-km) resolution weekly product for the 1985–2009 period [51]. This product uses only night-time retrievals, consistent with NOAA's Coral Reef Watch (CRW) thermal stress products. Data were excluded if deemed of poor quality (quality value <4, [52]) or if individual pixels were masked as land. Missing data were filled with temporal interpolation for gaps of 3 weeks or less, beyond which gap filling may produce unrealistic temperature values based on the time-dependent variability of oceanic processes. Remaining gaps were filled by comparing ambient temperatures in adjacent pixels with the spatial pattern of climatological temperatures, setting the gap-value to match this identified pattern.

Island-specific SST time series data were produced by spatially averaging the individual 4-km pixels that were intersected by or contained within the 30-m bathymetric contour for each island. Several metrics were then derived to quantify thermal variability on coral reefs. Monthly climatological means were first calculated for each location from the weekly data. The maximum monthly mean (the warmest of the 12 monthly values) served as the upper climatological range limit for each island, while the minimum monthly mean (the coldest of the 12 monthly values) provided the lower climatological range limit. Long-term mean SST was calculated by averaging all weekly data over the 25-year time series.

The ***HotSpot*** metric, developed by CRW, quantified the occurrence and magnitude of positive SST anomalies; i.e., SST values that exceeded the upper climatological range limit. The ***ColdSpot*** metric, developed here as an analogue to the HotSpot metric, quantified negative SST anomalies; i.e., SST values that were below the lower climatological range limit.

*Waves*: NOAA's Wave Watch III (WWIII; http://polar.ncep.noaha.gov/waves) is a global, full spectral wave model. We used WWIII one-degree spatial resolution, 3-hour output of mean significant wave height, dominant period, and direction from 1997 to 2010. Wave data were extracted from the one-degree grid cell in which each island location was located. Wave energy flux in kilowatts per meter (kW m-1) was calculated for each time step for the full time series. Wave energy flux (henceforth referred to as wave energy) is shown in the following equation:
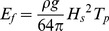
 where *ρ* is the density of seawater (1024 kg m-3), 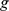
is the acceleration of gravity (9.8 m s-2), *H_s_* is mean significant wave height, and *T_p_* is the dominant wave period. Although wave height is frequently used in ecological research and is often easier to contextualize, wave energy (given its dependence on wave period and wave height) is a more realistic estimate of wave forcing, and, therefore, a more ecologically relevant parameter with which to quantify wave impact [22], [53].

Several wave metrics were developed to encapsulate the spatial and temporal patterns in wave forcing. Using the native 3-hour WWIII output, daily maximum and minimum wave energy values were calculated for each island over the entire time series. Climatological values were then calculated by taking the maximum and minimum values over a 5-day temporal window, and then averaging these values (separately) for the same 5-day period over all years. A 5-day temporal window was chosen for these calculations because it captures the episodic nature of wave events and avoids averaging out the signal of potentially heterogeneous data. Upper and lower climatological range limits were obtained by taking the highest and lowest values from the maximum and minimum wave energy data sets, respectively. Long-term mean wave energy was calculated by averaging all 3-hour output values over the 14-year time series.

To quantify when wave forcing fell outside the climatological range, a novel ***wave anomaly value (WAV)*** metric was calculated by identifying all days that were above or below the upper and lower climatological range limits from the respective daily time series. Analogous to the HotSpot and ColdSpot metrics previously defined, the WAV metric quantified the occurrence and magnitude of anomalous wave events at each island.

*Chlorophyll-a and Irradiance*: Remotely sensed ocean color algorithms are calibrated for optically-deep waters, where the signal received by the satellite sensor originates from the water column without any bottom contribution. In our study region, optically-deep waters are typically deeper than 15 – 30 m [48]. In optically-shallow waters such as lagoons, regions within atolls, and most coral reef environments, bottom substrate properties and sediment suspension may affect light propagation, which increases marine reflectance and data quality issues when quantifying in-water constituents, such as chlorophyll-*a* [54].

Satellite-derived irradiance, specifically photosynthetically available radiation (PAR; defined as downwelling irradiance between 400 and 700 nm), is subject to similar data quality concerns. The data production algorithm [55], in addition to a number of other quality control steps, incorporates irradiance attenuation in the overall calculation of irradiance. Attenuation sources in the atmosphere include the absorption and scattering of irradiance as a result of concentrations of ozone, water vapor, and aerosols. Attenuation sources at the air-sea interface include reflection, associated with surface properties such as sea-surface roughness and levels of sea foam [55]. Optically-shallow areas are often wrongly interpreted as irradiance attenuation sources, thereby leading to spuriously low irradiance values [55].

Eight-day, 0.0417° (hereafter 4-km) spatial resolution time series of chlorophyll-*a* (mg m-3) and irradiance (Einsteins m-2 d-1; henceforth E m-2 d-1) products derived from Moderate Resolution Imaging Spectroradiometer (MODIS; http://modis.gsfc.nasa.gov/) were obtained for the July 2002 – May 2011 period. Taking into account the data-quality concerns described above, we developed a multistep masking routine to remove contaminated data pixels. Following Maina et al., 2011 [12], we used the 30-m contour as the cutoff for pixel inclusion; all pixels inshore of the 30-m isobath were identified (Fig. 2A) and removed from the data set prior to analysis (Fig. 2B). This step, however, is not sufficient to ensure error-free chlorophyll-*a* and irradiance data sets, because pixels outside the 30-m isobath may still contain biased information associated with optically-shallow waters. This occurs because data pixels are box-like in shape and are georeferenced at their center point; thus, information contributing to any single pixel value is collected up to one-half a pixel diagonal distance away. To address this, we created a data exclusion zone of one-half a pixel diagonal in length (0.0295° or ∼3.27 km) everywhere perpendicular to the 30-m isobath, with all pixels on or within this zone also removed from the data set (Fig. 2C).

To determine the spatial domain that best represented island-scale chlorophyll-*a*, a series of spatially expanding, non-overlapping data inclusion zones were created for each island, with the width of each zone set at half the pixel diagonal. At all island locations, chlorophyll-*a* concentrations were compared between zones by taking the long-term mean for each pixel and calculating the average (±standard error) of all pixels within a given zone. In total, 6 zones were analyzed at each island representing distances 3.27–6.54 km to 19.62–22.89 km away from the 30-m bathymetric contour (see Fig. 3 for example). At 35 of the 41 locations, the zone most proximate to the island (zone one: 3.27 – 6.54 km from the 30-m contour) showed the highest concentration of chlorophyll-*a*, thereby capturing the signal most indicative of the island. These same pixels were used to represent the irradiance data set.

A series of metrics were derived using the masked, spatially constrained, and quality controlled chlorophyll-*a* and irradiance time series data sets. Eight-day climatological values were calculated by averaging all same 8-day time periods from each year over all years. The maximum and minimum island-specific climatological values were used as the upper and lower climatological range limits of chlorophyll-*a* and irradiance. Long-term mean chlorophyll-*a* and irradiance values were calculated by averaging all data over the entire time series from July 2002 to May 2011 for each location.

Analogous to the thermal and wave energy metrics previously described, we quantified the magnitude and occurrence of chlorophyll-*a* and irradiance values above and below their respective climatological range limits. This created ***chlorophyll-a anomaly value (CAV)*** and ***irradiance anomaly value (IAV)*** metrics.

*Bathymetry*: Gridded multi-beam bathymetric data were collected during Pacific RAMP surveys and provided by the Pacific Islands Benthic Habitat Mapping Center (http://www.soest.hawaii.edu/pibhm).

### Data Manipulation and Statistical Analysis

Wave, chlorophyll-a, and irradiance data were analyzed and manipulated using Matlab R2010a (http://www.mathworks.com). SST data were analyzed using IDL v8.0 (http://www.exelisvis.com/language/en-US/ProductsServices/IDL.aspx). Thirty-meter depth contours were derived using ArcGIS 9.3. A one-way analysis of variance (ANOVA) with a post-hoc Tukey comparison test was used to test the difference in long-term means, climatological range limits and average annual anomalies for all regions and pair-wise for each of the 10-pair combinations. Principle Component Analysis (PCA) of normalized data was used to explore similarities in environmental forcings across all islands and regions. Similarity Profile (SIMPROF) analysis [56] was used to test the presence of island groupings within each of the environmental metrics used in the PCA. The SIMPROF analyses were based on 9999 permutations at the 1% significance level, resulting in a significance of p<0.0001. ANOVA and Tukey comparisons tests were conducted in Matlab. PCA and SIMPROF analyses were conducted in PRIMER V6 [57].

## Results

Climatological range limits and long-term means for SST, wave energy, chlorophyll-*a*, and irradiance along with associated metrics quantifying the magnitude and occurrence of anomalous events; HotSpot & ColdSpot, WAV, CAV and IAV, respectively, are shown for all islands from the Northwestern Hawaiian, Hawaíi, Mariana, Equatorial and Samoa regions (Figs. 4, 5, 6, S1, S2, S3, S4; Tables S1, S2, S3, S4, S5, S6). Anomaly metrics were calculated as an annual average and are presented as a percentage of time above (positive anomaly) or below (negative anomaly) the upper and lower climatological range limit (Figs. 5, S3 – S4). For example, at Kure Atoll, the lower climatological range limit in SST is 18.98 °C. In an average year (over the 25-year record), SST at Kure Atoll was anomalously cold (colder than 18.98 °C) 6% of the year, or roughly 3 weeks a year.

To effectively compare latitudinal changes in climatological range limits and anomalies, Figure 5 presents islands in decreasing latitude (from north to south) from left to right. Hawaíi Island, and Johnston and Wake Atolls, however, are oriented according to geographic proximity to a region as opposed to strict latitudinal orientation. Hawaíi Island is relatively close (48 km) to Maui, while Johnston Atoll is located 1,300 km southwest of Oahu; both locations are geographically aligned with the Hawaiian Archipelago. Wake Atoll is most proximate to the Mariana region and is therefore placed after Johnston Atoll and before Farallon de Pajaros. There is less than a 4° latitudinal deviation between the geographic based island sequence in Figure 5 and the actual latitudinal sequence of islands (see Table 1 for specific geographic coordinates).

### Climatological Ranges and Anomalies

*SST*: Long-term mean SST was significantly different among regions (F4,36 = 122.2, p<0.00001) except when comparing each of the Mariana, Samoa and Equatorial regions (p<0.05; Fig. 4A; Table S1). The Northwestern Hawaiian region was characterized by relatively low long-term mean SST (mean±se: 24.25°C±0.25), particularly at northern-most latitudes, while the Samoa region had the highest long-term SST (28.42°C±0.11).

Upper climatological range limits of SST differed between regions (F4,36 = 161.82, p<0.00001). Most regions were different from each other (p<0.05) with the exception of the Northwestern Hawaiian and Hawaíi regions, and when comparing each of the Mariana, Samoa and Equatorial regions (Figs. 5A, S1; Tables S1, S3). Across all regions, a Pacific-wide dichotomous pattern in upper climatological range limits was observed; the Northwestern Hawaiian and Hawaíi regions had similar upper limits (26.64°C±0.05 and 26.71°C±0.13, respectively), as did the Mariana, Equatorial, and Samoa regions (28.79°C±0.03, 28.48°C±0.21, 28.93°C±0.06, respectively). Jarvis Island (Equatorial region), an obvious outlier in the observed split pattern, showed an upper limit that was 0.82°C colder than the region mean. Lower climatological range limits also differed between regions (F4,36 = 67.3, *p<*0.0001), with all regions differing (*p<*0.05) except comparisons between the Mariana, Samoa, and Equatorial regions. Lower climatological range limits were coldest in the Northwestern Hawaii region (21.24°C±0.52) and increased with each subsequent region from Hawaíi (24.01°C±0.16), to Mariana (26.18°C±0.16), to Equatorial (27.19°C±0.17), to Samoa (27.24°C±0.17).

When comparing the climatological range across all islands, a narrowing pattern was observed from Kure (Δ7.46°C) to Jarvis (Δ1.14°C), and then a slight broadening pattern from Swains (Δ1.18°C) to Rose Atoll (Δ1.89°C). The observed variations in the climatological range were principally a result of variability in the lower climatological range limit.

Average annual HotSpots (positive SST anomalies) were significantly differently between regions (F4,36 = 21.57, p<0.00001), with the same regions having distinct average annual anomalies as had distinct upper climatological range limits in SST (p<0.05; Figs. 5A, S3; Tables S2–S3). The Equatorial region had the greatest average annual HotSpots (29.24%±5.03), followed by the Samoa (29.10%±1.13) and Mariana (28.75%±1.16) regions. HotSpots in the Northwestern Hawaiian (15.64%±0.39) and Hawaíi (14.37%±0.45) regions did not differ from each other but had much lower values (approximately half) and were significantly (*p<*0.05) different from HotSpots in the Equatorial, Mariana, and Samoa regions. Average annual ColdSpots (negative SST anomalies) also differed between regions (F4,36 = 75.25, *p<*0.0001) and were greatest in the Equatorial region (25.25%±2.47; *p<*0.05; Figs. 5A, S4; Tables S2 – S3). All other regions experienced comparatively low average annual ColdSpots (<11% of the year).

*Wave Energy*: Long-term mean wave energy was significantly different between regions (F4,36 = 68.93, *p*<0.0001) except between the Hawaíi and Equatorial regions, and when comparing Samoa with the Mariana and Equatorial regions (p<0.05; Fig. 4B; Table S1). The Northwestern Hawaiian region was characterized by the highest long-term wave energy (41.97 kW m-1±1.08), while the Mariana region had the lowest long-term wave energy (19.84 kW m-1±0.77).

Upper climatological range limits in wave energy were significantly different between regions (F4,36 = 105.23, *p<*0.0001), with the greatest upper limits observed in the Northwestern Hawaiian region (309.36 kW m-1±16.16; *p<*0.05; Fig. 4b; Tables S1, S4). The northern three islands within the Northwestern Hawaiian region; Kure (370.18 kW m-1±73.48), Midway (367.42 kW m-1±71.74) and Pearl and Hermes Reef (364.45 kW m-1±68.37), had the greatest upper climatological range limits; ∼2.5 times greater than islands within the Hawaíi region (e.g., Oahu 151.12 kW m-1±21.43) and 5–7 times greater than all other islands in this study. It should be noted, however, that the Mariana and Samoa regions are located in areas of the Pacific that experienced tropical cyclones on an annual to interannual basis, which generated positive WAV values that were 10 times greater or more than the regional average. Lower climatological range limits in wave energy were also significantly different between regions (F4,36 = 80.59, *p<*0.0001), with all regions differing from each other (*p<*0.05) with the exception of the Northwestern Hawaiian and Hawaíi regions, and the Equatorial and Samoa regions.

Average annual positive WAVs (positive wave anomalies) were significantly different between regions (F4,36 = 38.39, *p<*0.0001) but were generally low across all regions (<5%; Fig. 4b; Tables S2, S4). Average annual negative WAVs (negative wave anomalies) were also significantly different between regions (F4,36 = 21.42, *p<*0.0001) and were generally greater (5 – 11% of the year at all locations) than positive WAVs (Figs. 5B, S4). Thus, in an average year, there were more days with anomalously low wave energy than days with anomalously high wave energy.

*Chlorophyll-a*: Long-term mean chlorophyll-*a* was significantly different between regions (F4,36 = 59.55, *p*<0.0001) with the exception of the Mariana and Samoa regions and when comparing Hawaíi with the Northwestern Hawaiian and Samoa regions (p<0.05; Fig. 4C; Table S1). Long-term mean chlorophyll-*a* was greatest in the Equatorial region (0.1672 mg m-3±0.0175); between 1.8–3.4 times greater than long-term chlorophyll-*a* in each of the other regions.

Upper climatological range limits in chlorophyll-a were also significantly different between regions (F4,36 = 50.01, p<0.0001). Most regions differed from each other (p<0.05) with the exception of Hawaíi and Samoa, and Mariana and Samoa (Figs. 5C, S1; Tables S1, S5). The Equatorial region had the greatest upper climatological range limit (0.2350 mg m-3±0.0175, p<0.05), with Jarvis (0.2913 mg m-3±0.0757), Howland (0.2432 mg m-3±0.0213), and Baker (0.2410 mg m-3±0.0493) characterized by the greatest upper climatological range limits of all study locations. Kure Atoll (0.206 mg m-3±0.0362), the northern-most island of the study, had an upper climatological range limit approximately equal to the upper limit at Palmyra Atoll (0.186 mg m-3±0.0083 mg m-3) and Kingman Reef (0.213 mg m-3±0.005), despite the 22° of latitude or ∼2,420 km separating Kure (28.42 °N, -178.33°W) from Palmyra (5.88°N, -162.09°W) and Kingman (6.39°N, -162.38°W). Lower climatological range limits were also significantly different between regions (F4,36 = 19.32, p<0.0001), with most regions differing (p<0.05) with the exception of Northwestern Hawaiian and Hawaíi, and Samoa when compared to the Hawaíi and Mariana regions. The Equatorial region not only had the greatest upper climatological limit in chlorophyll-a, but also had the greatest lower climatological limit (0.1120 mg m-3±0.0237 mg m-3, p<0.05). The islands in this region therefore experience relatively high annual productivity when compared to the other study locations.

Average annual positive CAVs (positive chlorophyll-*a* anomalies) were not significantly different (F4,36 = 0.94, *p = *0.45; Fig. 4c; Tables S2, S5) and were low across all regions (5.50 – 9.12%). Average annual negative CAVs (negative chlorophyll-*a* anomalies) were generally greater (7.58 – 15.29%) and were significantly different between regions (F4,36 = 5.06, *p = *0.0024), with the Hawaíi and Samoa regions differing, and the Equatorial region differing from all regions except the Northwestern Hawaiian region (*p<*0.05).

*Irradiance*: Long-term mean irradiance values differed between regions (F4,36 = 2.96, *p = *0.0325), although the only paired-wise comparison that differed was between the Northwestern Hawaiian and Equatorial region (p<0.05; Fig. 4D; Table S1). Both across and within the regions, long-term mean irradiance showed little spatial heterogeneity with the exception of the Equatorial region, which had both the highest (50.1 E m-2 d-1) and lowest (39.05 E m-2 d-1) island mean irradiance values.

Upper (F4,36 = 14.11, *p<*0.0001) and lower (F4,36 = 19.71, *p<*0.0001) climatological range limits in irradiance were significantly different between regions (Figs. 5D, S1, S2; Tables S1, S6). Across all islands in the Northwestern Hawaiian, Hawaíi and Mariana regions, the climatological range exhibited a narrowing pattern with latitude, ranging from Δ33.75 E m-2 d-1 at Kure to Δ18.08 E m-2 d-1 at Guam. In addition, the Equatorial region contained islands with the lowest upper climatological limits (Kingman, 45.11 m-2 d-1±1.08; Palmyra, 46.72 m-2 d-1±1.03) as well as islands with the highest lower climatological range limits (Jarvis, 42.36 E m-2 d-1±0.94; Howland, 43.12 E m-2 d-1±2.04; Baker, 44.20 E m-2 d-1±2.69) when compared to all other islands in each of the regions.

Average annual positive and negative IAVs (positive and negative irradiance anomalies, respectively) were significantly different between regions (F4,36 = 19.88, *p<*0.0001 and F4,36 = 11.51, *p<*0.0001, respectively; Figs. 5D, S3 – S4; Tables S1, S6). Average annual positive IAVs increased with each subsequent region from Northwestern Hawaiian to Samoa; however, only the Northwestern Hawaiian, Hawaíi and Mariana regions when each compared to the Equatorial and Samoa regions were significantly different, with the former regions greater than the latter (p<0.05). When comparing islands across all regions, a majority (85%) of islands had higher average annual positive IAVs than negative IAVs.

### PCA and SIMPROF Results

PCA on upper climatological range limits indicated wave energy and SST as the major loadings of PC1 (59.3% of variation explained) and chlorophyll-*a* and irradiance as the major loadings of PC2 (26.2% of variation explained; Fig. 6A). PCA of upper limits resulted in islands clustering by region, with little overlap of islands among different regions. However, the SIMPROF analyses overlaid on the PCA (dashed lines) resulted in four distinct groupings (*p<*0.0001). Pearl and Hermes, Midway and Kure of the Northwestern Hawaiian region formed a unique group, whereas the remaining islands within the Northwestern Hawaiian region (Maro, Lisianski, Laysan, French Frigate Shoals, Necker and Nihoa) grouped with much of the Hawaíi region (Niihau, Kauai, Oahu, Molokai, Lanai, and Oahu). Hawaíi Island and Johnston Atoll grouped with islands from the Samoa and Mariana regions. All islands within the Equatorial region grouped together, the only geographic region to be statistically grouped together.

PCA of long-term mean values also indicated wave energy and SST as the major loadings of PC1 (53.8% variation explained) and chlorophyll-*a* and irradiance as the major loadings of PC2 (33.8% variation explained; Fig.6B). PCA of means clustered the Mariana and Samoa regions together, while islands from Hawaíi and Northwestern Hawaiian regions were less clustered, primarily separating along PC1. Jarvis, Howland and Baker clustered together, well removed from other islands. Kingman and Palmyra were clustered in proximity to islands within the Hawaíi region. The SIMPROF analysis indicated greater complexity of island clustering than inferred from the PCA, with a total of 7 distinct groups (*p<*0.0001). The Samoa and Mariana (save Wake) regions formed a group, as did islands from the Hawaíi region along with Wake and Nihoa, Necker, and French Frigate Shoals of the Northwestern Hawaiian region. Distinct groups also included: Howland, Baker and Jarvis (Equatorial); Kingman and Palmyra (Equatorial); Laysan, Lisianski, and Maro (Northwestern Hawaiian); Midway and Kure (Northwestern Hawaiian); and Pearl and Hermes (Northwestern Hawaiian).

Unlike the PCA results from upper climatological range limits and long-term means, a PCA based on the lower climatological range limits indicated irradiance and SST as the major loadings of PC1 (49.8% of variation explained) and chlorophyll-*a*, SST and wave energy as the primary loadings of PC2 (30.3% of variation explained; Fig. 6C). PCA of lower limits resulted in islands from different regions clustering in close proximity to each other. For example, Kingman, Palmyra and Johnston clustered with the Samoa region, while much of the Hawaíi region clustered with the Northwestern Hawaiian region. Jarvis, Howland, and Baker islands clustered well apart from all other islands, as did Pearl and Hermes, Kure and Midway Atolls. The SIMPROF analysis resulted in seven distinct groups (*p<*0.0001). The Mariana region was divided into three separate groups: islands geographically located from Guam to Sarigan; from Guguan to Farallon de Pajaros; and Wake Atoll. Islands from the Samoa region were grouped with Johnston (Hawaíi) and Kingman and Palmyra (Equatorial). Other lower limit groupings were: Jarvis, Howland and Baker (Equatorial); Pearl and Hermes, Midway and Kure (Northwestern Hawaiian); and Laysan, Lisianski, Maro, French Frigate Shoals, Necker, and Nihoa (Northwestern Hawaiian) with islands from the Hawaíi region (save Johnston).

## Discussion

Our analyses suggest considerable spatial heterogeneity in climatological ranges across U.S. Pacific coral reef ecosystems (Figs. 4, 5, 6, S1 – S2; Tables S1, S2, S3, S4, S5, S6). The emergent spatial patterns and the degree of variability in upper and lower climatological range limits of SST, wave energy, chlorophyll-*a* and irradiance were unique for each environmental forcing, with no obvious or clear common spatial patterns across forcings. For example, upper climatological range limits in wave energy followed an overall trend with latitude; islands located farther north (i.e., Kure and Midway) showed higher upper limits of wave energy compared to islands located farther south (i.e., Swains and Tutuila), with the change in wave energy between island locations reducing with decreasing latitude (Figs. 5B, S1). In contrast, the upper climatological range limit for chlorophyll-*a* concentration exhibited a complex spatial pattern that appeared to be influenced by a combination of region and island specific environmental forcings (Figs. 5C, S1). For example, Jarvis, Howland and Baker Islands are located on the equator and had the highest upper climatological range limits of all the islands examined. The upper climatological range limit at Jarvis Island, and presumably at Howland and Baker Islands, is driven by regional thermocline variability and by a unique localized upwelling phenomenon created by island-current interactions [19] that increase nutrient availability and enhance local productivity [58]. Kure Atoll was also characterized by a relatively high upper climatological range limit of chlorophyll-*a*, though this atoll is located 28° (3,080 km) to the north of Jarvis Island. Kure Atoll is likely influenced by local conditions, but also by proximity to the Transition Zone Chlorophyll Front (TZCF); a basin-wide chlorophyll front located at the boundary between the subtropical and subpolar gyres that migrates southward in the winter months to the latitude of the atoll [59]. Hence, the spatial patterns are unique for each environmental forcing.

The PCA and SIMPROF analyses revealed two important findings when comparing the environmental setting across the study region (Fig. 6). First, different environmental metrics can lead to differences in the environmental setting. For example, when comparing upper climatological range limits, Palmyra and Kingman Atolls had a similar environmental setting when compared to Jarvis, Howland, and Baker Islands (Fig. 6A). However, when comparing long-term means (Fig. 6B) and lower climatological range limits (Fig. 6C), Palmyra and Kingman grouped either independently or with islands from a different region entirely. Hence, careful consideration of the appropriate environmental metric must be made, as the choice of metric can have considerable bearing on interpretation of the environmental setting at individual island and atoll reef ecosystems. Second, geographic proximity was not a prerequisite for similarities in the environmental setting. When comparing long-term means (Fig. 6B), environmental conditions at geographically separate locations were found to be similar. For example, all locations within the Samoa region grouped with the Mariana region despite a ∼6,000 km distance between the 2 archipelagos. Conversely, environmental conditions at geographically proximate locations were found to be different. For example, in the SIMPROF analysis, Midway and Pearl and Hermes Reef grouped separately despite being separated by only ∼150 km.

We also find that the spatial scale in which environmental data are synthesized can substantially affect the quantification of environmental forcings. Previous research synthesized data on a 1 × 1° (∼12,100 km2) spatial grid [3], [13], [14] and/or grouped numerous islands across large ocean provinces [12], [13]. Our study encompassed 41 islands and atolls, many of which are completely isolated and are 1.13–8254 times smaller in area (land + reef area; Table 1) than a 12,100 km2 grid cell. When comparing expanding spatial footprints of data inclusion, we found that chlorophyll-*a* concentrations decreased at relatively short distances (>7 km; Fig. 3) from 85% of our study locations. Hence, proportionally scaling environmental data to the study location (as opposed to using large, spatial grids) is more likely to capture local environmental forcings that may be pertinent to coral reef ecosystem dynamics.

The spatial patterns in chlorophyll-*a* concentration observed here have important implications for coral reef research. Chlorophyll-*a* has been used as an indicator of stress inducing environmental conditions to coral reefs [12], serving as a proxy for eutrophic conditions, which can affect the thermal tolerance of corals [46], and as a proxy for increased turbidity and sedimentation, which can result in coral mortality [47]. In general, these studies are geographically specific and associate high levels of chlorophyll-*a* with poor water quality, attributable to anthropogenic influences. In this study, the islands with the highest chlorophyll-*a* concentrations; Jarvis, Howland, Baker, Palmyra and Kingman, are each uninhabited and are characterized by high hard coral cover and large numbers of predatory fishes [33], [60], [61]. Thus, natural elevated levels of chlorophyll-*a* may serve to positively influence coral reef ecosystems, possibly through increased food availability (i.e., phytoplankton) for primary consumers [62], or through enhanced nutrient levels, important for sessile benthic organisms such as corals and algae [63]–[66].

Assessing climatological conditions and the history of anomalies may provide insight into coral reef ecosystem response to future climate scenarios. For example, history of exposure to positive SST anomalies may serve to enhance physiological tolerance (i.e., acclimation) of corals to resist future bleaching [16], [43], [45] or possibly increase survival of corals through natural selection (i.e., adaptation) [67], [68]. Island and atoll reef ecosystems influenced by enhanced upwelling could possibly serve as refugia for coral reef ecosystems by mitigating the extent of future increases in ocean temperatures [18]. Also, reef ecosystems subject to a high annual wave energy may have increased susceptibility to coral breakage in the future [69], given projected changes in ocean chemistry and associated impacts to coral calcification [39].

Despite the broad applicability of the environmental forcing metrics presented here, we acknowledge that there are some limitations. First, environmental forcing can have considerable spatial heterogeneity on intra-island scales [31]. This intra-island variability is not captured in these mesoscale analyses. ‘Tuning’ these metrics to the intra-island scale is the focus of our subsequent research. Second, despite our attempts to produce metrics that are representative of environmental conditions on coral reefs, satellite-derived environmental data can differ from in-situ, reef level measurements [70]. Third, although we feel the use of deep-water wave information is a good, first-order approximation of wave forcing on coral reefs [71], waves are highly complex as a result of refraction, dissipation, and other wave-bathymetry interactions [22], leading to potential differences between nearshore wave forcing and the wave metrics presented in this research. Fourth, this research is limited to locations with bathymetric data at the resolution needed to clearly identify the 30-m contour. Future work will explore the feasibility of using remotely sensed, depth-flagged grid cells [12] to include a greater number of coral reef ecosystems throughout the Pacific. A final limitation pertains to climatological range calculations, as the data set lengths for irradiance, chlorophyll-*a* and waves are temporally limited (<15 years), hindering the calculation at time-scales that are likely relevant to biological adaptation.

Despite these limitations, this research provides an important environmental context for which to compare coral reef communities across the U.S. Pacific coral reef ecosystems. It is our hope that the results of this study will help to elucidate the differential importance of environmental forcings to coral reef communities in this region, fostering more targeted ecosystem research and aiding in the formulation of effective ecosystem-based management practices. It is also our hope this research will aid in the prediction of potential changes in coral reef ecology owing to a changing climate, an indisputable aspect of future reef system dynamics.

## Supporting Information

Figure S1 **Map representing upper climatological range limits in A) SST, B) wave energy, C) chlorophyll-a and D) irradiance across each of the regions that comprise the coral reef ecosystems of the U.S. Pacific.** Regions indicated in panel A are the same for panels B –D. Please see Figure 1 in main text as a reference for individual island and atoll locations.
(PDF)

Figure S2 **Map representing lower climatological range limits in A) SST, B) wave energy, C) chlorophyll-a and D) irradiance across each of the regions that comprise the coral reef ecosystems of the U.S. Pacific.** Regions indicated in panel A are the same for panels B –D. Please see Figure 1 in main text as a reference for individual island and atoll locations.
(PDF)

Figure S3 **Map representing average annual positive anomalies for A) SST, B) wave energy, C) chlorophyll-a and D) irradiance across each of the regions that comprise the coral reef ecosystems of the U.S. Pacific.** Anomalies are presented as a percentage of time, representing the average annual percentage of time above the lower climatological limit. Regions indicated in panel A are the same for panels B –D. Please see Figure 1 in main text as a reference for individual island and atoll locations.
(PDF)

Figure S4 **Map representing average annual negative anomalies for A) SST, B) wave energy, C) chlorophyll-a and D) irradiance across each of the regions that comprise the coral reef ecosystems of the U.S. Pacific.** Anomalies are presented as a percentage of time, representing the average annual percentage of time below the lower climatological limit. Regions indicated in panel A are the same for panels B –D. Please see Figure 1 in main text as a reference for individual island and atoll locations.
(PDF)

Table S1 **Summary table of climatological range limits and long-term means for SST, wave energy, chlorophyll-a and irradiance.** Values are presented for each of the 41 islands and atolls that comprise the coral reef ecosystems of the U.S. Pacific. Please see methods section in main text for more information pertaining to island- and atoll-scale climatology metric development.
(XLSX)

Table S2 **Summary table of average annual anomalies for SST, wave energy, chlorophyll-a and irradiance.** Positive and negative anomalies are presented for each of the 41 islands and atolls that comprise the coral reef ecosystems of the U.S. Pacific. Please see methods section in main text for more information pertaining to island- and atoll-scale anomaly metric development.
(XLSX)

Table S3 **Summary table of SST anomalies.** Anomalies are summarized by year and include the annual number and average annual magnitude of both positive and negative anomalies for each of the 41 islands and atolls that comprise the coral reef ecosystems of the U.S. Pacific. Please see methods section in main text for more information pertaining to island- and atoll-scale anomaly metric development.
(XLSX)

Table S4 **Summary table of wave energy anomalies.** Anomalies are summarized by year and include the annual number and average annual magnitude of both positive and negative anomalies for each of the 41 islands and atolls that comprise the coral reef ecosystems of the U.S. Pacific. Please see methods section in main text for more information pertaining to island- and atoll-scale anomaly metric development.
(XLSX)

Table S5 **Summary table of chlorophyll-** ***a*** **anomalies.** Anomalies are summarized by year and include the annual number and average annual magnitude of both positive and negative anomalies for each of the 41 islands and atolls that comprise the coral reef ecosystems of the U.S. Pacific. Please see methods section in main text for more information pertaining to island- and atoll-scale anomaly metric development.
(XLSX)

Table S6 **Summary table of irradiance anomalies.** Anomalies are summarized by year and include the annual number and average annual magnitude of both positive and negative anomalies for each of the 41 islands and atolls that comprise the coral reef ecosystems of the U.S. Pacific. Please see methods section in main text for more information pertaining to island- and atoll-scale anomaly metric development.
(XLSX)

Appendix S1 **Supplemental Materials and Methods.**
(DOCX)
