# Correction: Quantifying Climatological Ranges and Anomalies for Pacific Coral Reef Ecosystems

**DOI:** 10.1371/journal.pone.0251616

**Published:** 2021-05-06

**Authors:** Jamison M. Gove, Gareth J. Williams, Margaret A. McManus, Scott F. Heron, Stuart A. Sandin, Oliver J. Vetter, David G. Foley

In the Methods and Materials section, there is an error in the equation for Wave Power in the fifth paragraph. There is a missing squared term on gravity (g). Please view the complete, correct equation here:

$$E_{f}=\frac{\rho g^{2}}{64\pi }H_{s}^{2}T_{p}$$
