# Supplementary figures and images for: Quantifying Climatological Ranges and Anomalies for Pacific Coral Reef Ecosystems
Source: PLoS One. 2013 Apr 18;8(4):e61974. doi: 10.1371/journal.pone.0061974 (PMC3630142; doi:10.1371/journal.pone.0061974)

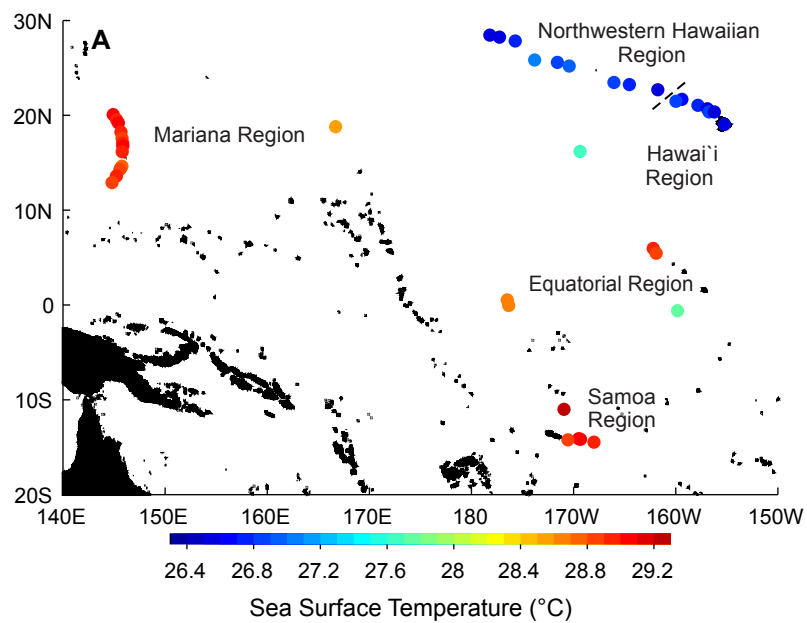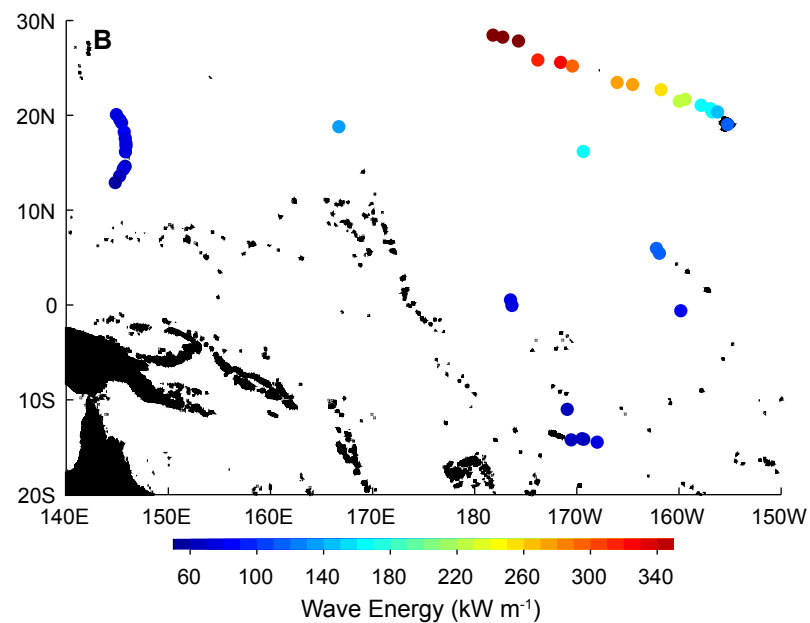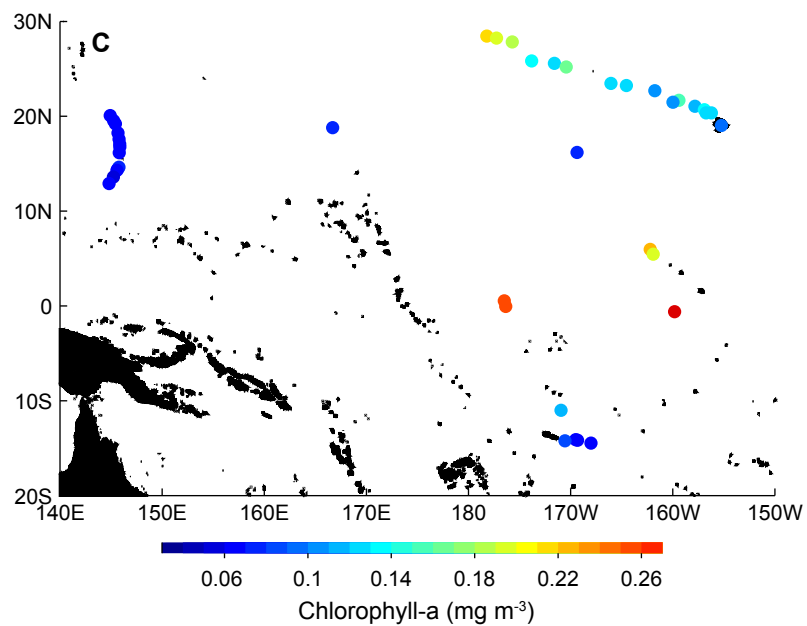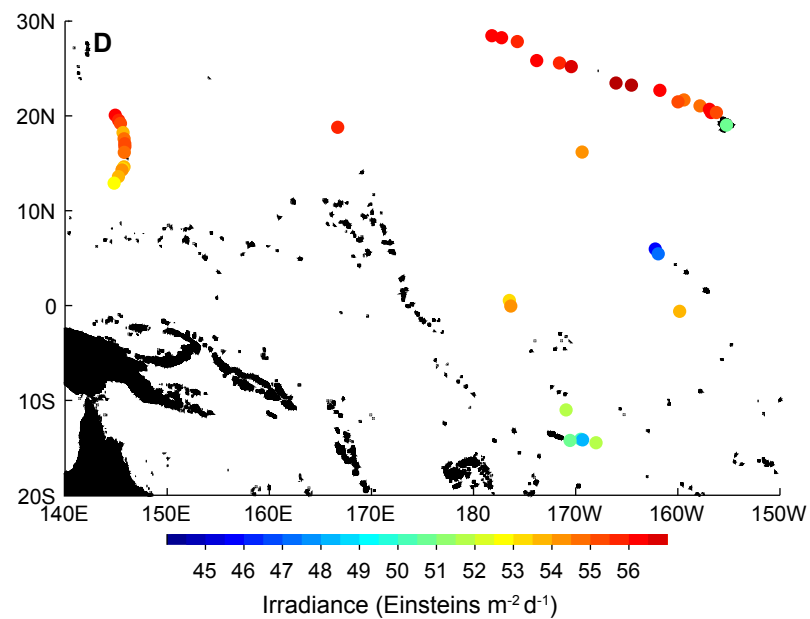

Supplement: Figure S1 — Map representing upper climatological range limits in A) SST, B) wave energy, C) chlorophyll-a and D) irradiance across each of the regions that comprise the coral reef ecosystems of the U.S. Pacific. Regions indicated in panel A are the same for panels B –D. Please see Figure 1 in main text as a reference for individual island and atoll locations. (PDF) [file pone.0061974.s001.pdf]

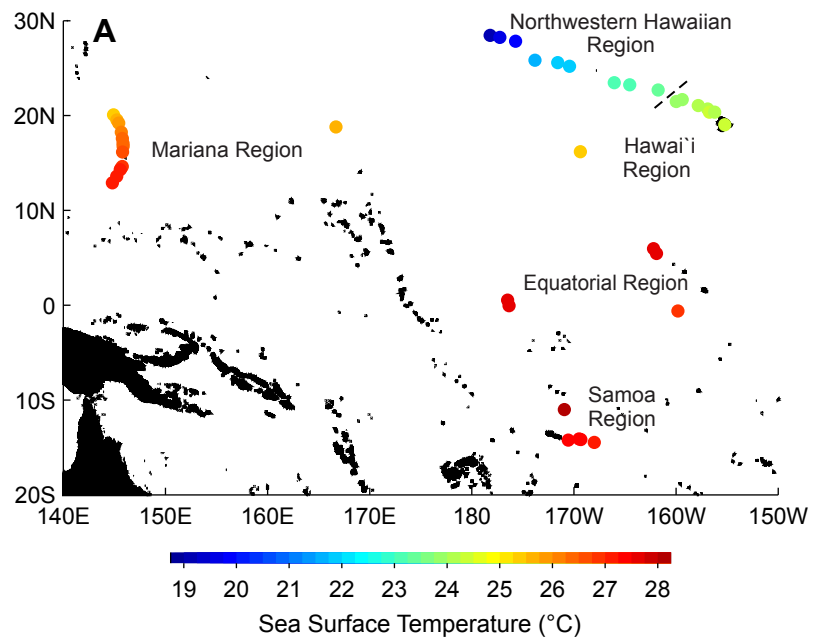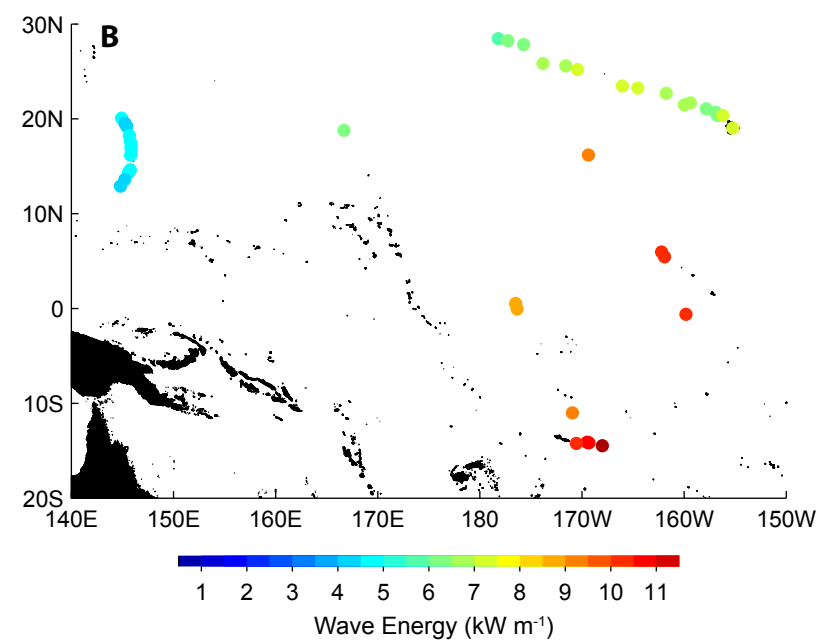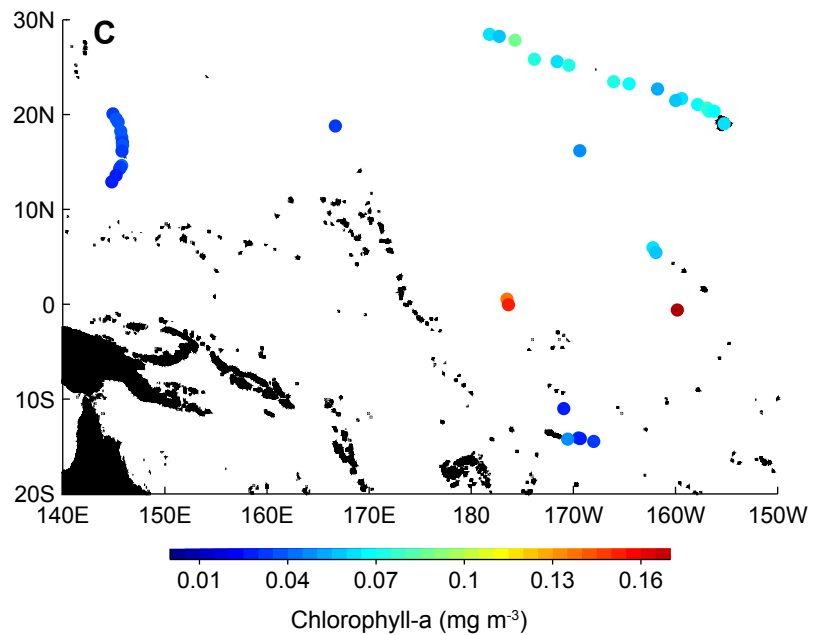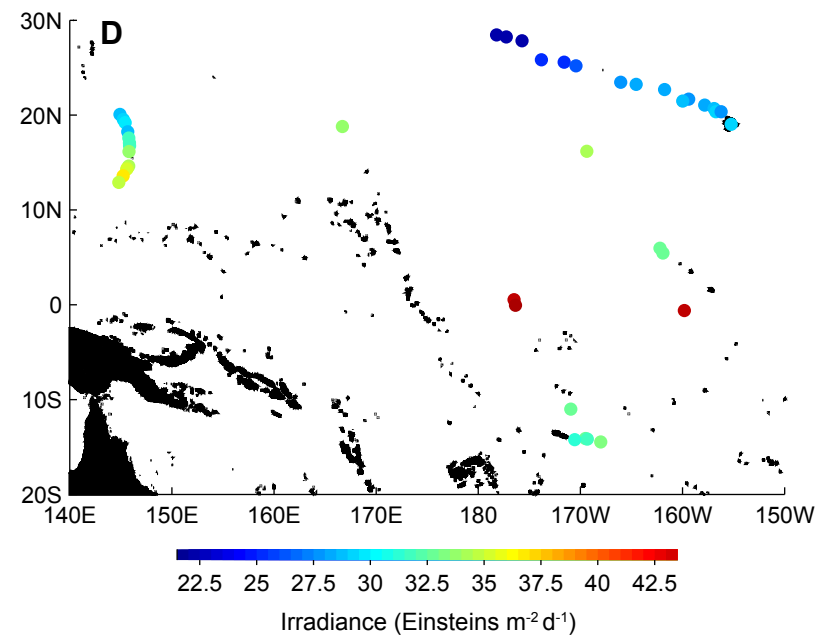

Supplement: Figure S2 — Map representing lower climatological range limits in A) SST, B) wave energy, C) chlorophyll-a and D) irradiance across each of the regions that comprise the coral reef ecosystems of the U.S. Pacific. Regions indicated in panel A are the same for panels B –D. Please see Figure 1 in main text as a reference for individual island and atoll locations. (PDF) [file pone.0061974.s002.pdf]

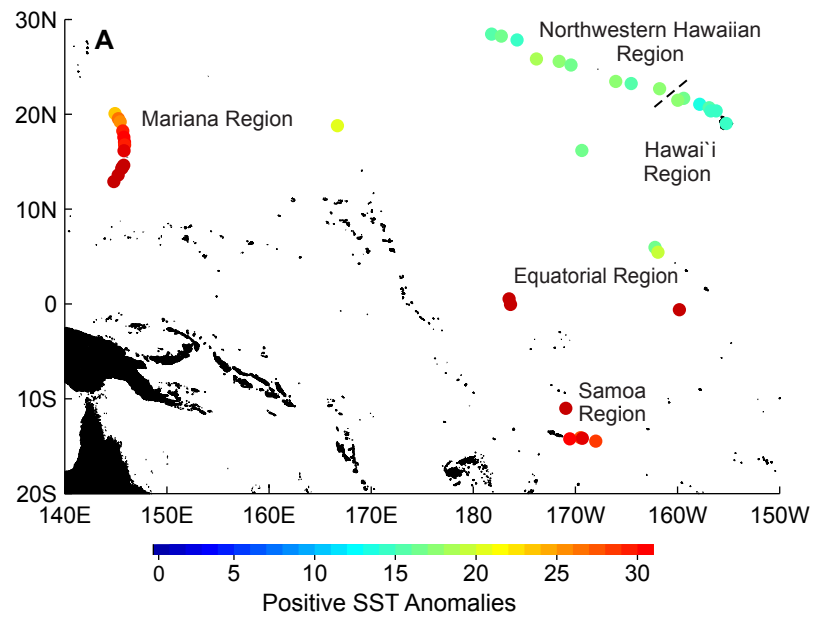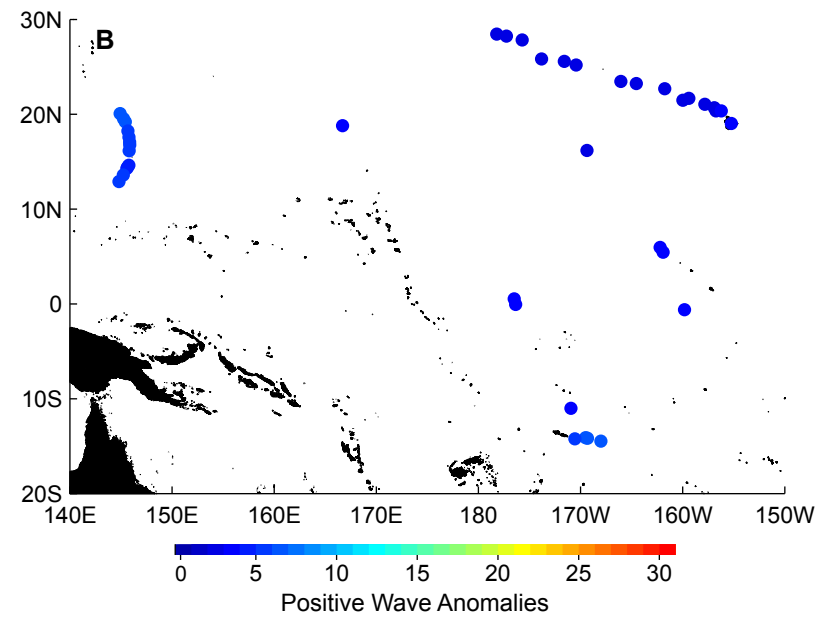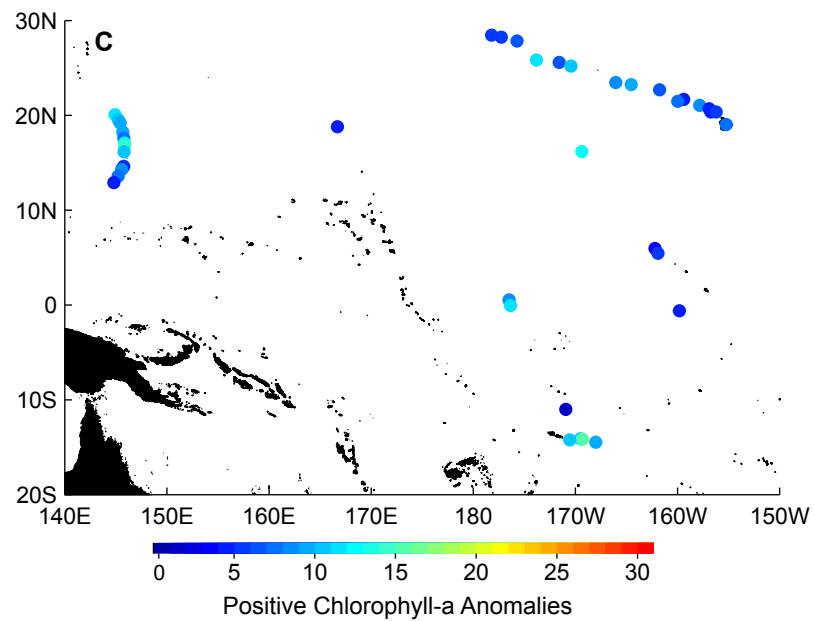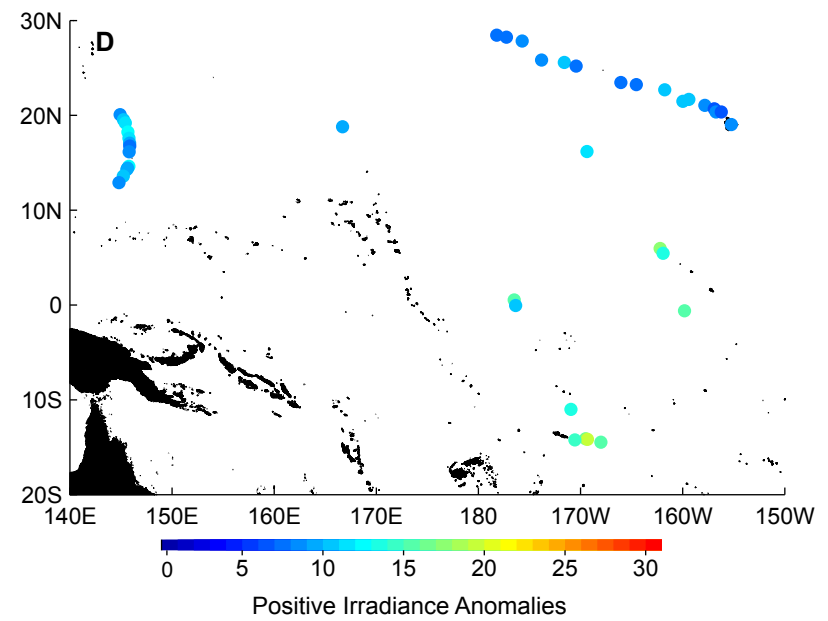

Supplement: Figure S3 — Map representing average annual positive anomalies for A) SST, B) wave energy, C) chlorophyll-a and D) irradiance across each of the regions that comprise the coral reef ecosystems of the U.S. Pacific. Anomalies are presented as a percentage of time, representing the average annual percentage of time above the lower climatological limit. Regions indicated in panel A are the same for panels B –D. Please see Figure 1 in main text as a reference for individual island and atoll locations. (PDF) [file pone.0061974.s003.pdf]

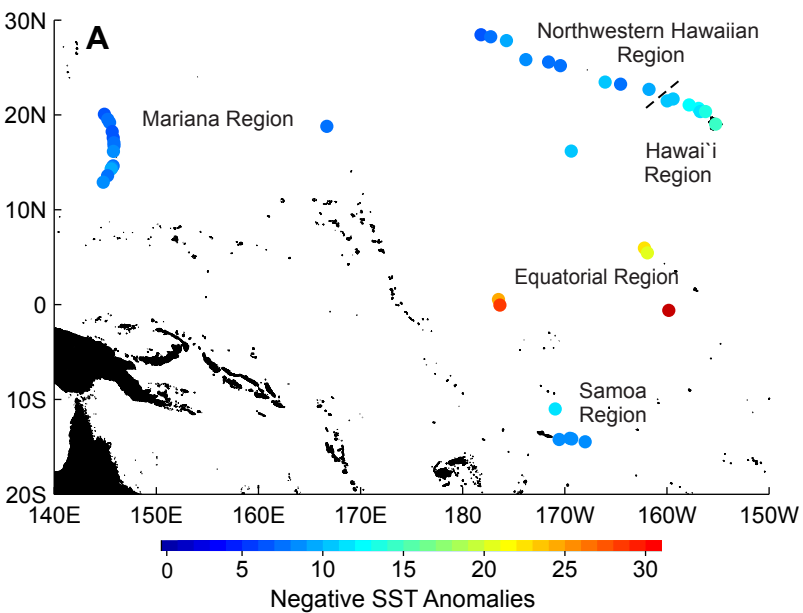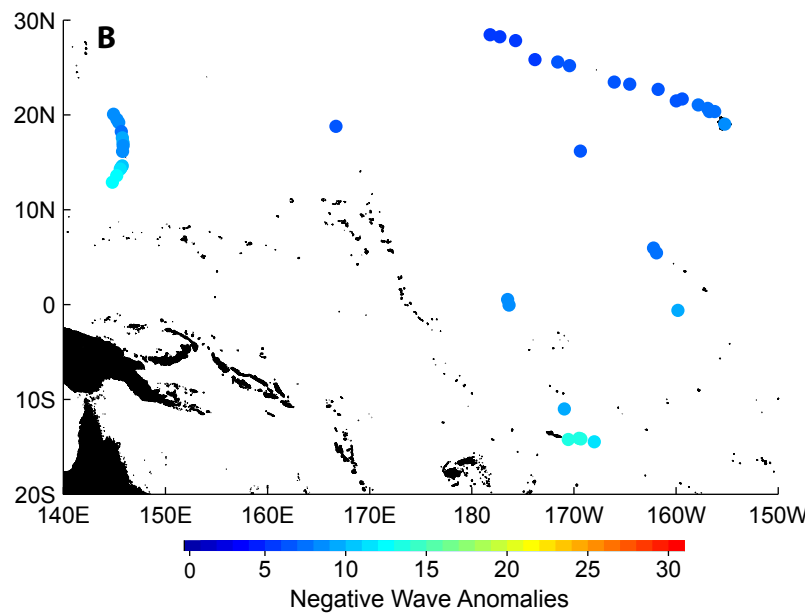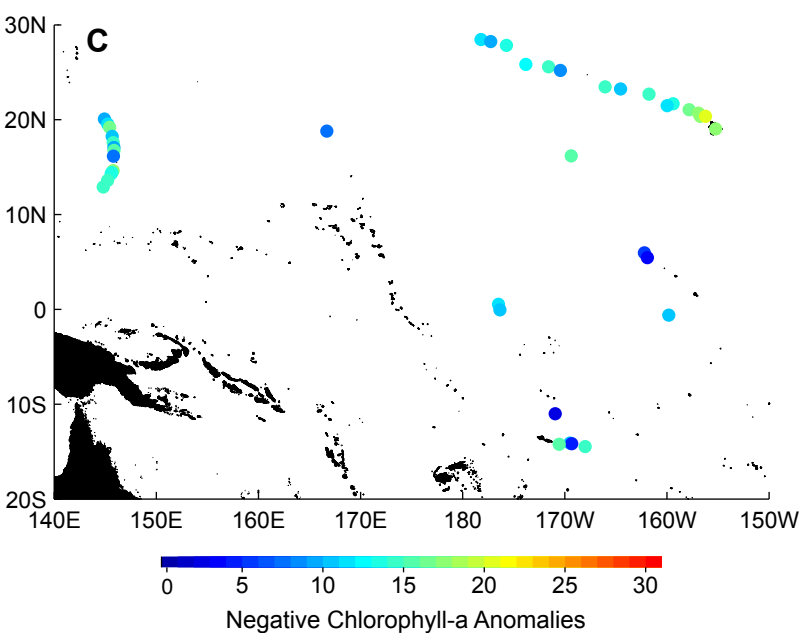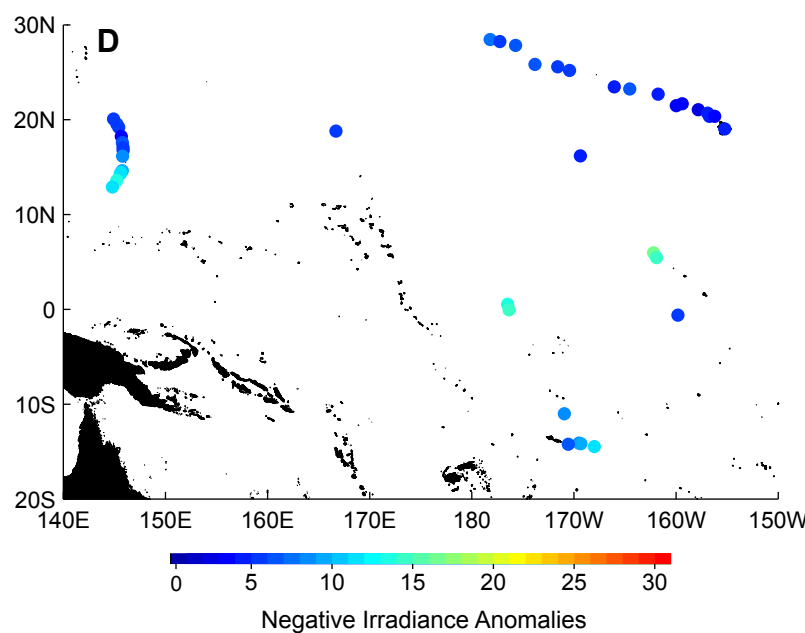

Supplement: Figure S4 — Map representing average annual negative anomalies for A) SST, B) wave energy, C) chlorophyll-a and D) irradiance across each of the regions that comprise the coral reef ecosystems of the U.S. Pacific. Anomalies are presented as a percentage of time, representing the average annual percentage of time below the lower climatological limit. Regions indicated in panel A are the same for panels B –D. Please see Figure 1 in main text as a reference for individual island and atoll locations. (PDF) [file pone.0061974.s004.pdf]
