# Supplementary material for: Quantifying Climatological Ranges and Anomalies for Pacific Coral Reef Ecosystems
Source: PLoS One. 2013 Apr 18;8(4):e61974. doi: 10.1371/journal.pone.0061974 (PMC3630142; doi:10.1371/journal.pone.0061974)
Supplement: Appendix S1 — Supplemental Materials and Methods. (DOCX) [file pone.0061974.s011.docx]

**Appendix S1: Supplementary Materials and Methods**

*SST climatological calculations*: SST climatology data used by NOAA’s Coral Reef Watch (CRW) for near real-time monitoring of thermal stress leading to coral bleaching is an average of the years 1985-1990 and 1993 (time center in 1988/89; [[1](#_ENREF_1)]). We elected to calculate the 4 km monthly climatology values using the years 1985-2006 for two reasons: (1) the source Pathfinder v5.0 data for 2007-2009 were processed only as “interim” values (as opposed to “final”) and, though likely small, we did not want any impact of this to affect the calculations for the finalized years; and (2) we wanted to take advantage of as many years as possible in creating the climatologies to ensure any bias from a short-term event (with respect to the dataset length) was minimized. However, the second of these sets the time center of the averaging to 1995/96 and would, in the event of any background trend within the data, alter the baseline from the established global monitoring system. To counter this, we calculated trends through the dataset to reset the climatology value to that corresponding to the time center in 1988/89, consistent with the procedure used by CRW in experimental product development.

*Chlorophyll-a quality control:* Given the relatively close proximity of these satellite-derived data to land (where present), potential sources of error remain in the chlorophyll data, either due to in-water constituents such as suspended sediment, atmospheric effects associated with clouds, or other unknown sources that could result in erroneous values. As an additional quality control step, data were examined to identify time periods in which chlorophyll-a values from an island-location were substantially greater (factor ≥ 2) when compared to values from the entire time series. Two time periods from two separate regions were identified. The first time period occurred in the Mariana region between July 4^th^ and 20^th^, 2003 in which chlorophyll-a concentrations were up to three times greater than the next highest value from the 8+ year data record at seven island locations (Sarigan, Guguan, Alamagan, Pagan, Asuncion, Maug and Farallon de Pajaros; Figure 1). This time period coincided with the volcanic eruption of Anatahan (Figure 1; Anatahan is not identified, but resides 40 km to the south of Sarigan), which began 11-May-2003 and continued for roughly two months, spewing ash and material up to 11,000 km into the atmosphere [[2](#_ENREF_2)]. Although regional changes in phytoplankton response were reported immediately following the initial eruption [[3](#_ENREF_3)], no in situ chlorophyll-a data were available to validate these findings, nor the values observed in our chlorophyll-a data set.

The second time period identified occurred between April 7^th^ and 15^th^, 2005 from two islands within the Samoa Region (Tau and Tutuila; Figure 1). Recorded chlorophyll-a concentrations from Tau and Tutuila were over 3 times greater than the next highest values. No typhoons within the previous month were recorded, and monthly precipitation data from Pago Pago, Tutuila, showed no anomalous rainfall prior to this period (source: National Weather Service, <http://www.prh.noaa.gov/hnl>)

These two events represent at most 0.95% of the total chlorophyll-a data from each of the islands. Given the elevated chlorophyll-a values associated with the geological event in the Mariana Region and the event of unknown origin in the Samoa Region, inclusion of these data in climatological calculations would result in inflated climatologic range limits that are likely unrepresentative of long-term chlorophyll-a conditions, and thereby misleading. However, until future work can definitively rule these data as invalid, we included these events in the anomaly metrics outlined in this research.

**References**

1. Skirving WJ, Strong, A.E., Liu, G., Arzayus, F., Liu, C., Sapper, J. (2006) Extreme events and perturbations of coastal ecosystems: Sea surface temperature change and coral bleaching. In: Richardson LL, LeDrew EF, editors: Springer Netherlands. pp. 11-25.

2. Trusdell FA, Moore RB, Sako M, White RA, Koyanagi SK, et al. (2005) The 2003 eruption of Anatahan volcano, Commonwealth of the Northern Mariana Islands: Chronology, volcanology, and deformation. Journal of Volcanology and Geothermal Research 146: 184-207.

3. Lin II, Hu C, Li Y-H, Ho T-Y, Fischer TP, et al. (2011) Fertilization potential of volcanic dust in the low-nutrient low-chlorophyll western North Pacific subtropical gyre: Satellite evidence and laboratory study. Global Biogeochem Cycles 25: GB1006.
